# An Overview of Deep Learning Methods for Left Ventricle Segmentation

**DOI:** 10.1155/2023/4208231

**Published:** 2023-01-30

**Authors:** Muhammad Ali Shoaib, Joon Huang Chuah, Raza Ali, Khairunnisa Hasikin, Azira Khalil, Yan Chai Hum, Yee Kai Tee, Samiappan Dhanalakshmi, Khin Wee Lai

**Affiliations:** ^1^Department of Electrical Engineering, Faculty of Engineering, Universiti Malaya, Kuala Lumpur, Malaysia; ^2^Faculty of Information and Communication Technology, BUITEMS, Quetta, Pakistan; ^3^Department of Biomedical Engineering, Faculty of Engineering, Universiti Malaya, Kuala Lumpur, Malaysia; ^4^Faculty of Science & Technology, Universiti Sains Islam Malaysia, Nilai 71800, Malaysia; ^5^Department of Mechatronics and Biomedical Engineering, Lee Kong Chian Faculty of Engineering and Science, Universiti Tunku Abdul Rahman, Malaysia; ^6^Department of Electronics and Communication Engineering, SRM Institute of Science and Technology, Kattankulathur, India

## Abstract

Cardiac health diseases are one of the key causes of death around the globe. The number of heart patients has considerably increased during the pandemic. Therefore, it is crucial to assess and analyze the medical and cardiac images. Deep learning architectures, specifically convolutional neural networks have profoundly become the primary choice for the assessment of cardiac medical images. The left ventricle is a vital part of the cardiovascular system where the boundary and size perform a significant role in the evaluation of cardiac function. Due to automatic segmentation and good promising results, the left ventricle segmentation using deep learning has attracted a lot of attention. This article presents a critical review of deep learning methods used for the left ventricle segmentation from frequently used imaging modalities including magnetic resonance images, ultrasound, and computer tomography. This study also demonstrates the details of the network architecture, software, and hardware used for training along with publicly available cardiac image datasets and self-prepared dataset details incorporated. The summary of the evaluation matrices with results used by different researchers is also presented in this study. Finally, all this information is summarized and comprehended in order to assist the readers to understand the motivation and methodology of various deep learning models, as well as exploring potential solutions to future challenges in LV segmentation.

## 1. Introduction

The capability of a machine to simulate and impersonate human intelligence processes is referred to as artificial intelligence. Machine learning is a subbranch of artificial intelligence which is based on the idea to enable machines or computers to perform without being specifically programmed. The machine can learn from data and focus on the use of the pattern and experience to improve the performance of the computer in making decisions on its own. In this way, the machine becomes capable of developing analytical models to adopt new situations autonomously.

Deep learning (DL) is a subfield of machine learning associated with a process inspired by the formation and function of the brain called an artificial neural network (ANN). DL is concerned with the interpretation of data based on the mechanism of the human brain by developing and simulating the algorithm worked on human brain analysis and learning. The training data are fed into the algorithm as input, and the successive layers of the DL algorithm analyse the original data to extract the features required for the targeted task. The training data is fed into the algorithm as input, and the successive layers of the DL algorithm analyse the original data to extract the features required for the targeted task. The entire process is free of human manipulation. One of the earliest practiced DL techniques is ANN with a deep network structure [1]. The multilayer perceptron models [2] have been proposed with the rapid progress in the research areas of computer vision (CV) and human brain neurons. This yields the development of other classical models such as back-propagation neural network models, convolutional neural network (CNN) models [3], bidirectional recurrent neural networks [4], transformers [5], long short-term memory (LSTM) [6], and deep belief network [7] models.

These research findings have significantly helped the expansion of DL architectures, flooring the way for its substantial level applications in numerous areas, especially in image processing. Image classification, image registration, object detection, and image segmentation were among the main tasks performed by the DL algorithms very efficiently.

These image processing methods were applied in various fields such as surveillance [8, 9], intelligent transportation system [10, 11], wireless communication [12, 13], web mining [14, 15], robotics [16, 17], civil [18], and the most important in medical image processing [19]. In medical, DL is used for the segmentation of various structures from the medical images [20], detection of different diseases [21–23], and also for image registration to have a better view of images [24].

The cardiac images are one of the medical images used for the assessment of patient health. Different cardiac images are used for the analysis of cardiac function. Assessment of cardiac function performs an essential part in medical cardiology for patient supervision, risk estimation, disease analysis, and therapy evaluation [25, 26]. For cardiac diagnosis, digital images are the basic tool used for the computation of subsequent clinical indices from the shape and structure of the heart. From the structure of the heart, the assessment of the left ventricle (LV), right ventricle (RV), and myocardium (MYO) are the main assessments. LV is one of the central issues and the attention of cardiac function study and disease diagnosis. Delineation of LV boundary is of great clinical importance for the study of heart parameters such as the ejection fraction (EF), stroke volume (SV), LV mass (LVM), end-systolic volume (ESV), and end-diastolic volume (EDV) [27].

Some studies have reviewed the segmentation of medical and cardiac images. However, to the best of the author's knowledge, those investigations did not investigate LV segmentation solely and explicitly. Keeping in mind the importance of LV, the primary focus of this research is to review only the segmentation of LV using DL models. This paper provides a comprehensive overview of different DL architectures used for the LV segmentation. This review has been carefully summarized to present the state-of-the-art DL algorithms focusing on the LV segmentation task. To find out the quality research in the area, the Web of Science database was used as a search engine. The keywords, “left ventricle”, “segmentation, and “deep learning” were used to find out the related papers. The articles which primarily do not emphasize LV segmentation were excluded because the scope of this review included an analysis of LV based segmentation. The review has been conducted using resources published between 2018 and onwards until December 2021.

**Figure 1:**
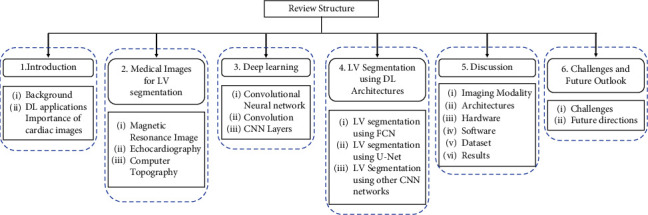
Article structure diagram.

In this article first, we discussed the three different imaging modalities used for the LV assessment in Section 2.  Section 3 presents the basics concepts related to DL and CNN. Different DL architectures used for the LV segmentation are reviewed in Section 4. The section is subdivided based on the different approaches used with DL such as preprocessing, deformable models, and clinical indices calculation. The discussion about the architecture, hardware, software, and datasets used for training, and evaluation matrices used to analyze the performance of models is presented in Section 5. The complete structure of the article is depicted in Figure 1.

## 2. Medical Images for LV Assessment

Different medical imaging modalities were used for the assessment of LV. These modalities include magnetic resonance images (MRI), echocardiography, computer tomography (CT) scan, myocardial perfusion imaging, multiple gated acquisition scanning, gated blood-pool SPECT, and fusion imaging. However, the most used imaging modalities in literature for LV segmentation are MRI, US, and CT scans. The detail of these images is presented in this section.

### 2.1. Magnetic Resonance Images

MR imaging is a widely used technique in the cardiac armamentarium. The official name is recognized as “cardiovascular magnetic resonance (CMR),” when the MRI is employed on the heart or cardiovascular system. Its diagnostic precision has preceded it to become the gold-standard for heart analysis [28].

MRI is suitable for the evaluation of heart chambers [29, 30], size, and blood flow through major vessels [31], heart valves [32], and pericardium [33]. In addition, for LV size and mass measurement, the MRI is considered a reference standard [34, 35]. Its adaptability is incomparable to other different imaging methods. It provides not only precise anatomic information but also gives functional information that helps in finding patients at risk. Three-dimensional geometric analysis of the LV by CMR provided more appropriate information about the shape of the LV than the traditional echocardiography with high fertility and low variability [36]. CMR has also been successful in observing LV hypertrophy in patients with apparently normal echocardiographic results [37].

**Figure 2:**
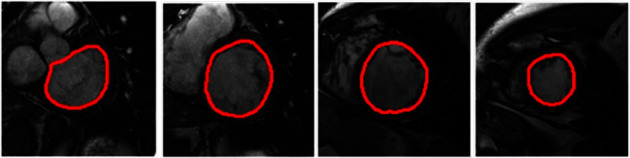
Four examples of LV boundary in CMR images.

Besides these outstanding outcomes, a few important limitations of CMR need to be remembered. It faces problems with costs, limited availability, and lack of portability. These constraints prevent the use of CMR normally. Compared with other imaging modalities, CMR inspections are very costly and inadvisable for patients with metallic implants such as graft stents and cardiac pacemaker devices. Cardiac MRI may not be accessible immediately in all centers, and it can be a difficult instrument to work out in patients who require serial monitoring. Another condemnation of CMR is the period of examination to acquire LVM data. It also has some minor issues such as device incompatibilities and patient tolerance.  Figure 2 shows LV segmentation in MRI images. The red area is LV segmented using a CNN. [38].

### 2.2. Echocardiography

Echocardiography used high-frequency ultrasound waves to produce anatomical images of the heart. That is why it is referred to as ultrasound (US) imaging. It is the largely used imaging modality for the examination of cardiovascular diseases [39]. Due to its easy accessibility, outstanding temporal resolution, real-time imaging, and noninvasive nature, the US is considered the basic imaging for measuring the LV function. US has become the primary preference for the analysis of LVM. A regular LVM calculation is an essential part of the US examination [40]. US imaging is also used in measuring the decrease in LVM after the treatment [41].

**Figure 3:**
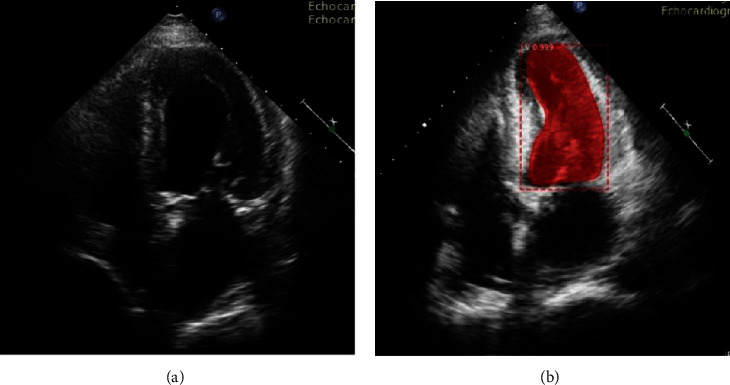
US image (a) and its US image with LV boundary (b).

The most used US imaging is two-dimensional US (2DE) and three-dimensional US (3DE). An example of 2DE is shown in Figure 3 and the LV boundary is shown by the red line. Although M-mode US imaging is also in use, due to different limiting factors of M-mode such as it only identifies the function of the basal segment while 2D and 3D can perform the whole LV segmentation, the use of M-mode is very limited. Using the 2DE, LVM can directly be calculated. Similarly, by attaining the pyramidal image, we can look at the 3-dimensional image of the whole heart. The 3D imagining of heart anatomy can be obtained using 3DE and it has also overcome the limitation of 2D imagining. Therefore, 3DE has gained considerable importance over 2DE and M-mode in various patient populations [42]. Inadequately, 3DE is not commonly available and costly compared to 2D imagining. The other limitation of the US imaging is the speckle noise [43] and low contrast ratio [44], which limits its performance.

### 2.3. Computer Topography

A computed tomography (CT) scan of the heart provides a cross section of the structure of the heart. It characterizes the X-ray attenuation features of tissues being imaged [45, 46]. CT is a growing imaging method for the noninvasive computation of heart anatomy and function. LV size and mass estimation can be computed using the CT modality. CT is also found as a good alternative for the LV size and mass calculation for those patients who have contraindications to CMR [42]. The study in [47] compares CT and US and also finds that CT can be used as an alternative to the US.

**Figure 4:**
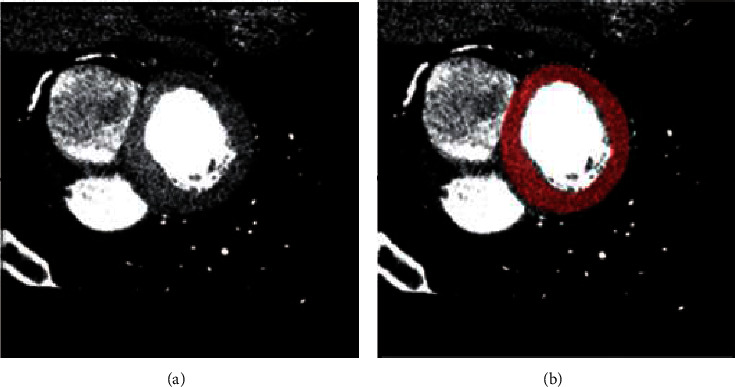
CT scan of LV (a) and LV boundary (b).

Though CT has many advantages, a few constraints are not ignorable. CT cannot be employed as real-time intraprocedural assistance due to unavoidable ionization radiation exposure called a stochastic effect [48]. Repetitive regular use can raise the cancer risk. The increase in image quality results in a higher dose of radiation. The left part of Figure 4 is a CT scan of the heart and in the right part, the LV is highlighted. [49].

## 3. Deep Learning

**Figure 5:**
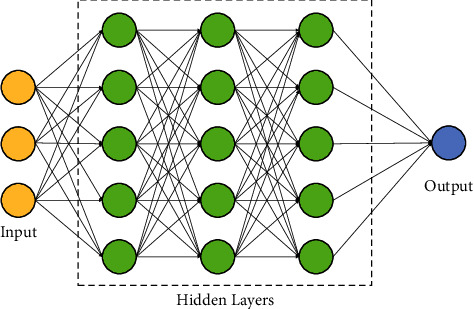
A general architecture of a deep neural network with three hidden layers.

DL can be defined as a machine learning algorithm that deals with neural networks. Neural networks with a deep structure or with more than 2 hidden layers are also referred as deep neural networks. A general architecture of DL is shown in Figure 4. DL is a representation learning (subtype of machine learning) with multiple levels of representation [50]. For the past several years, DL has been developed as a popular tool that attracts the attention of researchers from several fields. It helps to overcome the weaknesses of traditional methods and solve complex problems to achieve better results. The popularity of DL is doable due to large datasets, computational performance, training techniques (ReLu), and advanced networks (CNN). With the increase in databases, DL has exponentially achieved success both in commercial and academia. Not only the software base advancement help DL to achieve success, the latest hardware such as graphical processing units (GPU's) improved the ability of DL [51]. Deeper layers improve the system's experience by learning the features from data and making complex structures deeper and simple [52]. Therefore, it is a novel discovery for solving problems in those areas which has high dimensional data. Inspired by brain function, deep neural networks are built from many hidden layers sandwiched between the input and output layers. The general architecture of a deep neural network is presented in Figure 5.

### 3.1. Convolutional Neural Network

DL architectures are performing excellently in solving traditional artificial intelligence problems. The most established, progressive, and widely used is CNN. The following section discusses CNN, its variants, and its applications.

Among all the models of neural networks, CNN is the most dominant approach to solving problems of CV. The idea of CNN architecture was developed in the 1980s [53] but due to the lack of computational ability of hardware, high processing machines, and large storage devices to deal with big images, the idea did not flourish. The concept accelerated as the processing of machines increased in terms of computation and database to retrieve and store. Later in [54], CNNs were successfully applied in classification problems and performed brilliantly in CV applications. The gradient-based learning algorithm highly motivated CNN to produce optimized weights. CNN performed far better than other multi-layered perceptrons. The CNN weights are shared and are not needed to learn again for the same object at different locations. It recognizes visual patterns, directly from raw image pixels. This decreases the number of learnable parameters. CNN performance is impressive on 2D and 3D images. CNN model has minimized the preprocessing task and the back-propagation learning method improved the performance as it has provided a solution to deal with nonlinearity with the decrease in computation process due to a smaller number of weights. CNN has been producing better results in object recognition, behavior recognition, audio recognition, detection, recommendations localization, classification, and segmentation tasks.

#### 3.1.1. Convolution

Convolution is a mathematical operation that involves the multiplication and addition (weighted average) of two functions. The first function (*x*) represents input data and the second function (*w*) represents kernel and together they produce the output, called feature maps. CNN is similar to neural networks that use weights and biases. It involves a convolutional layer in the neural network that applies to input data of an acceptable type. The CNN architecture is divided into two divisions: feature extractors and classifiers. Each convolution layer finds specific features from the input data using a shared weight called kernels, and with *n* number of kernels, the convolution layer determines *n* features. The input of each layer is the result of the previous layer. A simple CNN consists of a convolutional layer, pooling layer, rectifier unit, fully connected layer, and classifier. The convolutional layer is a building block of CNN. The input image convolves with the kernel (learnable filter). The kernel slides over the input image and the size of the kernel are somehow learned from the input image. Some parameters drive the size of output i.e., depth, stride, and padding. The CNN compresses the fully convolutional network by lessening the connections and sharing the weight of the edge.

**Figure 6:**
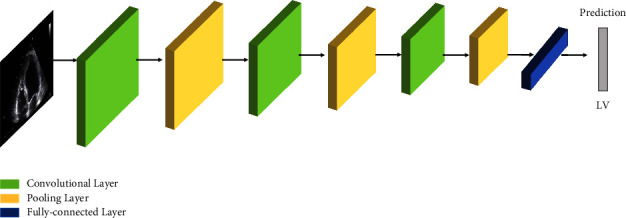
General structure of deep convolutional neural network.

Figure 6 shows a general CNN structure; the input image is convolved with kernels to extract the features. The result of convolution is then passed through the pooling layer (mostly Max pooling). The CNN extracts the features using these layers and finally a fully connected layer [55] gives the predicted output.

Convolution performs 3 main tasks: sparse interaction, parameter sharing, and equivariant representation.

- Sparse interaction: In a neural network, every output unit interacts with every input having separate parameters. These parameters help to determine the relationship or interaction between the input and output units. CNN uses kernels of different sizes which are smaller than input data in size. This reduces the number of learning parameters and the storage space and increases computation efficiency.
- Parameter sharing: It uses the same parameter for more than one chunk. In convolution, each kernel value is used at every point of input other than boundary values. It helps CNN to use only one set instead of multiple parameters for every location. It reduces the storage requirement further.
- Equivariance: It refers to the shift in the feature map by the same amount as the input shifts. Convolution does the same but not naturally [50].

#### 3.1.2. CNN Layers

For the past few decades, CNN is performing intensely in CV (detection, recognition, tracking, estimation, processing, analysis, learning, restoration, and reconstruction) as the popular machine learning algorithm. The GPUs have also brought extra efficiency in their results. The boost CNN gain is through several factors such as a large labeled training dataset, rectifier linear unit, regularization (dropout), and augmentation. The strength of CNN is extracting discriminative features at different levels. The CNN architecture consists of a convolutional layer, a nonlinearity layer, and a polling layer followed by a fully connected layer.

(i) Input layer: This layer understands the input data. It gives the contents of input data and has no learnable parameters. So, this layer has nothing to do with learning.
(ii) Convolutional layer: Convolutional layer performs convolution operation which is the trademark of CNN architecture. This layer holds learnable parameters such as weights and biases. This layer contains filters or kernels, used to detect edges, shapes, and patterns of the given input image. Kernels are convolved with each input feature/image pixel to produce feature maps as an output. A dot product between each input and filter is performed, followed by summing each dot product output, and finally, a bias is added. Bias can be configured according to network requirements. The convolutional layer reduces the computational cost by reducing the input size:
  (1) $$\begin{array}{c} Z_{i}^{l}=\overset{K^{l-1}}{\underset{J=1}{\sum }}W_{i,j}^{l}*Z_{j}^{l-1}+B^{l}\text{.} \end{array}$$
The kernel computes the product of weight and input of kernel size. It also determines the desired features based on kernel weights. Equation (1) shows the operation of the convolutional layer, where *Z*_*i*_^*l*^ and *Z*_*i*_^*l*−1^ are the outputs of the current layer and previous layer, respectively, and *W*_*i*,*j*_^*l*^ and *B*^*l*^ represent filters and biases. Each neuron need not be connected to all other neurons in the preceding and following layer. The input is convolved with filters to produce an output where bias is added for nonzero value. The final output goes through a nonlinear activation function which activates the feature maps and forwarded the result to the next layer.
(iii) Pooling layer: The other name of this layer is the subsampling layer. This layer reduces the dimensions through downsampling operation. Average (uses the average value) and Max (uses the highest value) pooling are the two most used operations. The following subsampling function represents a pooling operation:
  (2) $$\begin{array}{c} Z_{i}^{l}=Sub-Sampling\left(Z_{j}^{l-1}\right)\text{.} \end{array}$$
(iv) Nonlinearity layer: It applies the relevant nonlinear activation function. The most common functions are sigmoid, rectified linear unit (ReLU), hyperbolic function, and SoftMax:
  (3) $$\begin{array}{c} a^{l}=f\left(Z^{l}\right)\text{.} \end{array}$$
(v) Dropout: This layer regularizes the CNN model, decreases computation, and increases the generalization. It randomly drops the units by assigning the zero weight to a set of units. This layer helps to avoid the overfitting problem.
Fully connected layer: This is a flattened layer with each neuron of the previous layer connected to each neuron of the current layer. Each neuron has a separate weight for each connection. This layer has the highest number of learnable parameters. The input data are linearly processed, passed through a nonlinearity, and then propagated to the next layer.

## 4. LV Segmentation Using DL Architectures

CNN has performed several CV tasks effectively and precisely, that is why it is a widely used DL technique for image segmentation, especially for medical images. In Section 4.1, several CNN architectures are reviewed which are used for the LV segmentation.

### 4.1. LV Segmentation Using Fully Convolutional Network

The fully convolutional network (FCN) [56] introduces the fully convolutional layers instead of fully connected layers. Therefore, FCN can handle the variable size of images and fewer parameters to be learned which also make the network faster. The FCN and its variants used for LV segmentation are explained below.

#### 4.1.1. FCN with Pre/Postprocessing

A three-step (preprocessing, LV segmentation, and postprocessing) LV segmentation method is proposed in [57]. In the first phase, LV is localized using the over feat algorithm [58] to determine the region of interest (ROI) which is then fed to the next phase where the segmentation is performed using a temporal FCN (T-FCN) architecture. The CNN model is pretrained with GoogLeNet and fine-tuned using LV images. The T-FCN adds another hidden layer at the decoding path to restore the original size. The segmented LV boundary is further refined in the third phase by using one of the two algorithms: fully connected conditional random fields (CRFs) with Gaussian edge potentials [59] and semantic flow [60]. To train the network, the TWINS-UK database was used which consists of more than 12,000 images. The result showed that T-FCN with CRF performs better segmentation and achieved the dice similarity coefficient (DSC) value of 0.9815, average perpendicular distance (APD) of 6.2903, and conformity index of 0.9610. This work only focused on the LV segmentation.

One of the preprocessing methods is to crop the ROI first and then apply the segmentation to the selected ROI. This procedure for LV segmenting is presented in [61]. The clinical parameters such as LV volume (LVV), LVM, SV, and EF were also analyzed by estimating the size of LV from MRI images. The class imbalance challenge was tackled by first finding out the ROI using an FCN model. A new FCN model was applied to these ROI images for LV segmentation. Class entropy and radial distance were used as loss functions. The model is trained and tested using two datasets: the Automatic Cardiac Diagnosis Challenge (ACDC) 2017 publicly available dataset and a local dataset. The ACDC-2017 dataset consists of 150 patients' data, while the local dataset consists of almost 6000 images. The performance is evaluated using the DSC and Hausdorff distance (HD) for cross-entropy loss and radial distance loss. The model is analyzed for both datasets and achieves almost the same results, which yield that the model is generalized and applicable to any dataset. The proposed model attained better DSC and HD values than that of U-Net and ConvDeconv-Net. The DSC value for LV segmentation of the proposed model on the local dataset is 0.95 and ACDC dataset is 0.94. Similarly, the HD value is 9.31 and 11.21 for the local and ACDC dataset, respectively.

#### 4.1.2. Improved FCN for Clinical Index Calculation

Chen Qin et al. [62] proposed a model that consists of two branches: the motion estimation branch and the segmentation branch. The unsupervised Siamese style recurrent spatial transformer network is utilized for motion estimation and FCN is used for the segmentation. Motion estimation is an unsupervised method that combined the motion estimation and segmentation layer which can also be referred to as a weakly supervised model. A total of 220 short-axis view subjects were obtained from a UK biobank study. The LV segmentation is assessed by separately segmenting the LV and also by combining the two models. The DSC value of 0.9217 is achieved for only segmentation, while 0.9348 is achieved for the combined model which depicts that the model performs better in combine mode.

Similarly, the LVV is calculated using MRI images in [63]. The volume of LV is a very important feature to evaluate the patient's cardiac health which requires LV segmentation. The method segments the LV for diastolic and systolic to calculate the volume of LV. The Sunnybrook dataset is used to train and test the model. Data augmentation is also applied by rotating the slice in different directions. The method used a local binary pattern in cascade to detect the ROI. Then, a CNN model is used to score the ROI and select the one with the maximum score. Finally, LV is segmented using hypercolumn FCN (HFCN) from the ROI. The HFCN features from different levels were concatenated to form a new layer, and segmentation was based on this new layer. The volume is calculated using both manual and HFCN. The variance estimation method is used to estimate the final prediction. This algorithm ranked fourth in the Second Annual Data Science Bowl competition organized by Kaggle. Although this algorithm performs very well in segmentation, still sometimes the model generated the irregular shape of LV as it does not use the prior knowledge of the 3D shape of LV.

The feasibility and accuracy of FCN to segment the scar tissues in LV were analyzed in [64]. The modified version of FCN, efficient neural network (ENet), is applied to cardiac images. The proposed network consists of 13 convolutional layers with a 3 × 3 kernel size and stride of 2, while a parametric rectified linear unit (PReLU) was used as an activation function. Cross-entropy was used as a loss function. The two protocols, protocol-1 and protocol-2, were used for the segmentation. In protocol-1, the ground-truth and original images were directly fed to the network for training and segmentation. Whereas, in protocol-2, the desired LV area was cropped before training the network. The images were cropped using Hough transform [65]. The dataset consists of 250 images of 30 patients which is further increased to 2000 images by applying the data augmentation technique. Protocol-1 and protocol-2 achieved the accuracy of 95.97% and 96.83%, the sensitivity of 97.31% and 87.89, the specificity of 68.77% and 88.07%, and the DSC value of 0.54 and 0.71, respectively. The result demonstrated that protocol-2 performs better than protocol-1, which depicts that cropping the ROI gives better results in segmentation.

#### 4.1.3. Loss Functions and Optimization Algorithm

Until now, we have explained the FCN models and their performance based on preprocessing or by applying some changes in the model. Nevertheless, one very important parameter is the loss function. In [66], the updated model of FCN with different loss functions was analyzed. An iterative multipath FCN (IMFCN) segmentation model for LV, RV, and MYO from MRI images was proposed. To tackle the class imbalanced problem, searching for ROI was performed and images were cropped using the method proposed in [67]. The proposed model consists of an encoder, feature fusion, decoder, and deep supervision. Encoder part used *s* [*i*] current slice, previous slice *s* [*i* − 1], adjacent slice *s* [*i* + 1], and already predicted segmentation *M* [*i*] as input. These features are inserted into the encoder part to extract new features. The new features are fed to the feature fusion which consists of different convolution layers of different sizes. The output features from the fusion block are fed to the decoder block which has unsampled features. Finally, the deep supervision part performs the segmentation. The efficiency of the model is also compared using different loss functions. Batch-wise class reweighting mechanism and batch-wise weighted dice loss function were utilized for multiclass segmentation. The results of the proposed models were evaluated and compared with U-Net and LVRV-Net. To quantitatively evaluate the performance, three metrics: DSC, average symmetric surface distance (ASSD), and HD were employed. Batch-wise weighted dice loss function shows the best results among all loss functions. In this research, the most inaccuracies in segmentation have occurred in apical and basal slices. Additional processing mechanisms can lessen these errors.

The focal loss was analyzed with the four skip connections in the FCN model [68]. The model was referred to as the focal residual network (FR-net). RestNet50 was used as a backbone network. Cross-entropy loss was calculated across the predicted probability and labeling. Focal loss was applied to improve preliminary segmentation results. Sunnybrook dataset was used. DSC and APD were used to evaluate the performance. The model results were also compared with U-Net and FCN and other work based on the Sunnybrook dataset. The models attained the DSC value of 0.93 and APD of 1.41.

In addition to the loss function, the optimization algorithm is also a key feature of the CNN model. In [69], the performance of optimization factors was analyzed for the CNN model. Six different optimization algorithms, namely, stochastic gradient descent (SGD), nesterov accelerated gradient [70], RMSProp [71], Adam [72], AdaDelta [73], and AdaGrad [74] were implemented to train CNN model. A CNN model was proposed and trained separately using all six optimization factors. Sunnybrook dataset was used for training and testing. The best performance was obtained by the RMSProp optimization technique. The model achieved the DSC of 0.93, APD 2.13, and percentage of good contour (GC) 95.64 using RMSProp optimization.

#### 4.1.4. Other FCN Techniques

One key limiting factor in DL is the amount of data. Training the FCN model using a large dataset for LV segmentation is studied in [75]. The FCN model is trained on the UK Biobank dataset. The images of 5,008 subjects (93,500 images) were used to train the model after data augmentation. The data were manually annotated by eight different experts. DSC, mean counter distance, and HD values of 0.94, 1.04, and 3.16 were achieved, respectively. The segmented LV is also used to measure the LV-EDV, LV-ESV, and LVM.

Another technique to enhance the model performance is to take advantage of the pretrained model. A pretrained VGG model (trained on ImageNet) was combined with FCN called FCN-all-at-once-VGG16 [49]. The model used skip connections to combine the hierarchical features from convolutional layers with different scales. Adam was used as an optimizer with an initial rate of 10^−4^. A dataset of a total of 1100 subjects was used by splitting the dataset of 100 subjects into 50 training, 30 validations, and 20 tests images. The next 1000 cases (diastolic) are segmented using a trained model and compared by 1000 manually drawn by an expert technician. The manual drawing was performed using the in-house software (A-view Cardiac, Asan Medical Centre, Seoul, Korea). For quantitative analysis, sensitivity and specificity evaluation matrices were used. The method is limited when the number of pixels of background (i.e., image pixels other than the LV mask) is large. The model was evaluated using four performance indices, i.e., DSC, Jaccard similarity coefficient, mean surface distance (MSD), and HD.

FCN was also used with a graph matching algorithm. The motion estimation of LV from MRI images was studied in [38]. The method consists of four steps: (1) endocardial contours of the LV were predicted using a FCN, (2) features of points in short-axis cine MRI were extracted using an FCN feature descriptor, (3) the correspondence between contours of the LV myocardium was estimated by a novel graph matching algorithm, and (4) the correspondence between two LV contours and the LV motion field was estimated using the FCN feature descriptor into the graph matching algorithm. The Medical Image Computing and Computer-Assisted Intervention (MICCAI) 2009 challenge database and the 33 subject's database [53] were employed to evaluate the proposed method.

**Figure 7:**
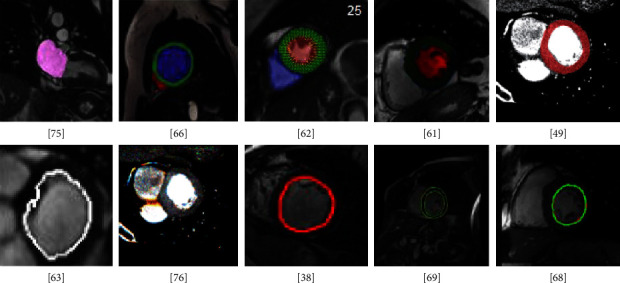
Sample of segmented images using FCN.

Consequently, FCN and its modifications show very decent results for LV segmentation. To enhance the performance different preprocessing and postprocessing techniques, loss functions, and data variability can be used.  Figure 7 illustrates examples of segmented images generated by FCN models.

### 4.2. LV Segmentation Using U-Net

In medical images, the required area to be segmented consists of a small area of the entire image. The U-Net [76] has shown substantial results in the segmentation of medical images. This is possible due to the ability of U-Net to continuously suppress the background region in training and emphasis the required areas that need to be segmented. That is why the most used network for LV segmentation is U-Net and its modification models.

#### 4.2.1. U-Net with Pre/Postprocessing

Studies applied the postprocessing or preprocessing with DL methods to yield good results. Guo et al. investigated the postprocessing effect on MRI images in [77]. Input Cardiac MRI is fed to U-Net and then labeling probabilities were generated. For the postprocessing Kernel cut, a segmentation technique was used. The output of U-Net is the input of continuous kernel cut which segments the desired part. LV, MYO, and RV were segmented using this approach. The result shows that, with less training data, reasonable segmentation results can be achieved.

The postprocessing on vivo diffusion tensor CMR was performed in [78]. A five-layer U-Net architecture is used to perform the LV segmentation followed by image registration. This helps to remove the bad images, and then, the final segmentation was applied. To increase the size of the dataset, data augmentation was used (translation and rotation). Batch normalization was used with U-Net to avoid overfitting. The model achieved a 0.93 median value of DSC.

The approach in [79] performs the preprocessing on the images by selecting the ROI by using the SinMod method. The ROI contains the desired part of the heart that is fed to the U-Net model for training. The curriculum learning (CL) strategy was utilized as a training strategy. The proposed methods were compared together with U-Net without CL, FCN (with and without CL), and hybrid gradient vector flow snake. The DSC, overlap, and mean average distance (MAD) were used as evaluation matrices.

In [80], the labeled images from the Kaggle database were used before training. The concept of transfer learning is utilized by pretraining the 3D U-Net model using Harvard data. The U-Net model was used to segment the LV, MYO, and RV. The DSC value achieved for LV segmentation was 0.87 without transfer learning and 0.95 using transfer learning.

The determination of ROI makes the segmentation task simple and accurate as the targets area is reduced to ROI instead of a complete image. This strategy was used in [81] and proposed for the three U-Net-based models. The proposed CNN architectures classify myocardial tissues and detect LV-ROI before LV quantification. For this experiment, the Sunnybrook cardiac dataset and the Cardiac Atlas Project (CAP) were used, which consists of 45 and 95 cases, respectively. Three new CNN architectures were proposed which are based on U-Net. The main purpose of the proposed models is to quantify the LV. Before LV quantification, LV-ROI detection and myocardial tissue classification were performed using the same U-Net architectures.

In the first proposed model, the encoding path comprises two 3 × 3 convolution operations, batch normalization, and residual learning. The 2 × 2 Max-pooling operation with stride 2 was performed after the residual learning [24]. The second and third proposed architecture is named as uInception and uXception. The network complexity was reduced in these networks. The SGD was used as an optimization factor and Jaccard distance as a loss function. The data augmentation was applied to increase the data size from 4,048 to 20,000. The segmentation accuracy was measured using the DSC and achieved 0.870, 0.869, and 0.868 for the proposed networks, respectively. Mean square errors of 0.0135, 0.0136, and 0.0138 were achieved while the mean absolute error was 0.0137, 0.0136, and 0.0138. Furthermore, EDV, ESV, SV, LVM, and EF were calculated as clinical indices.

#### 4.2.2. U-Net with Deformable Model

The combination of DL and deformable models as postprocessing can be combined to segment the LV. Veni et al. trained the U-Net model for LV segmentation from the A4C chamber view of US [82]. The segmented output is further refined using the deformable model. Using this technique, high accuracy is achieved by training the model with a very fewer amount of data, i.e., 69 images. The DSC value of 0.86 ± 0.06 was achieved.

#### 4.2.3. Improved U-Net for Clinical Index Calculation

Many studies focus on the calculation of various heart parameters such as EF, global longitudinal strain, or LVM, and for measurement of these parameters, LV segmentation is one of the primary tasks to be performed. A study was performed to validate that the DL methods can be used in real-time software that streams images directly from an ultrasound scanner [83]. A U-Net model was utilized for LV segmentation. The main goal was to calculate ventricular volume, EF, and mitral annular plane systolic excursion (MAPSE). All these parameters were based on the segmentation of LV. The accuracy of the model was evaluated by Bland–Altman analysis. The dataset of 75 patients was used and a value of (−13.7 ± 8.6)% for EF and (−0.9 ± 4.6) mm for MAPSE was achieved for Bland–Altman. The results show that DL is a feasible solution for the real-time calculation of clinical indices used for cardio analysis.

Similarly, LV segmentation was also performed to measure the GLS. The work in [84] utilizes the standard U-Net architecture and performs the four tasks on US images: (i) classification of cardiac view, (ii) segmentation of LV from the cardiac view, (iii) estimates of the regional motion, and (iv) a fusion of measurement. The segmentation architecture comprises five levels of upsampling and downsampling. All levels consist of two convolution layers with filters ranging from 32 to 128. The 3 × 3 filter size, 2 × 2 Max pooling, and 2 × 2 equal stride were utilized. Dice was used as a loss function and Adam as an optimizer.

A method to achieve LV segmentation based on temporal area correlation was proposed in [85]. U-Net was used as a base CNN model, and then, the multitask module is utilized for epicardium and endocardium segmentations. The output of the multitask module was fed to recurrent neural network (RNN). The RNN performs the temporal area correlation optimization. The average DSC of 0.90 ± 0.05 and average HD of 7.6 ± 4.5 was achieved. The LVM and EF have also been calculated to cross-validate the results.

For the quantitative analysis of the LV, segmentation is performed before quantification of LV parameters (area and dimension) [86]. The segmentation provides the structural information of LV which is further used for quantification. Initially, U-Net architecture was used as a segmentation model. Furthermore, a Deep-CQ segmentation model was proposed for LV segmentation that comprises the proposed loss function. The binary classification of each pixel as LV or background was performed using the Gibbs distribution function [87]. The segmentation performance was evaluated using DSC matrices and achieved 0.893 ± 0.05 value for Deep-CQ models, while U-Net yields 0.897 ± 0.041. The main object of this research work is the quantification of LV, and the Deep-CQ model performed better than U-Net for quantification while U-Net performed better than the Deep-CQ model in segmentation.

Estimation of myocardial perfusion is an essential step to measure the blood flow through the heart muscle. The arterial input function (AIF) extraction is an important phase for calculating the myocardial perfusion. The AIF estimation is highly dependent on detecting the LV size accurately. The LV segmentation to measure the AIF was performed in [88]. A U-Net model based on RestNet was designed to segment the LV. RestNet consists of batch normalization, ReLU, and convolution layers. To estimate the output probability, sigmoid or SoftMax was used. The kernel size used was 3 × 3 with 1 stride and 1 padding in all convolution layers. A weighted sum of cross-entropy and IoU was used as a loss function. To find out the best hyperparameters, 45 training sessions were performed and the best hyperparameters were used for final training. The labeling of LV and RV was performed using an ad hoc algorithm and experts cross-check the labeling. The model was trained using two different sets of classes: (i) LV and background and (ii) LV, RV, and background. The model achieved DSC values of 0.87 ± 0.08 for three classes and 0.82 ± 0.22 for two classes. The performance of the model trained for three classes was better than two classes because the contextual information extracted from three classes improves the LV segmentation performance.

From an entire echo cine, automatic LV segmentation was performed in [89]. The US images and optical flow of US images were first fed to the temporal window. The optical flow was calculated by the Horn–Schunck algorithm. The output of US image and optical flow US images act as input to the two separate encoder parts of U-Net. The output of both the U-Nets was concatenated. In the third part of the model, the concatenated data were passed to the bidirectional LSTM. The U-Net decoder finally up-sampled and segmented the LV. The data of 563 patients were used with a training and testing ratio of 80 and 20, respectively. Dice was used as a loss function and Adam as an optimizer. Network performance was compared with U-Net and U-Net Bi-Conv LSTM using the DSC. The model U-Net optical Bi-Conv LSTM achieved the best DSC value of 0.936 and accuracy of 0.977.

#### 4.2.4. Comparison of Different U-Net Models

The comparison of three well-known CNN architectures was performed by [90]. U-Net, wide U-Net, and U-Net++ were trained using the data of 94 patients. Data augmentation was used to increase the data size and to avoid the overfitting problem. The U-Net has 32, 64, 128, 256, and 512 feature maps, while the wide U-Net has 35, 70, 140, 280, and 560 feature maps. U-Net++ has an additional block of feature maps and skip connections. Exponential linear units (ELU) were used as an activation function in all layers except the last layer, where sigmoid was used. The model was trained using the original dataset and augmented dataset and the performance was assessed. The U-Net++ model performance was the best among the three models using an augmented dataset. The highest DSC value of 92.28 is obtained. Moreover, U-Net++ was less overfitted than U-Net and wide U-Net.

#### 4.2.5. U-Net Performance Based on Dataset Properties

Although the comparison among different variants of U-Net was performed in [90], the training dataset and data variability also affect the performance of the network. The effect of training datasets from different variability on the performance of the CNN model was analyzed in [91]. U-Net architecture was used as a segmentation model and assigned the names CNN1, CNN2, and CNN3 based on the training dataset variability. Three different training sets were collected for this research experiment. CNN1 was trained using the data from single center and single vendors with 25,389 images. CNN2 was trained by the set consisting of images from multiple centers by the single vendor and 27,488 images, while multiple centers and multiple vendor data were used to train the CNN3 model with 41,593 images. The training images were preprocessed for normalizing the resolution, cropping the images to 256 × 256, and normalizing the signal intensity. APD was used as an evaluation metric. CNN3 had the largest number of training samples and the highest variability, and it has achieved the best performance on unseen heterogeneous testing data with the highest value of 1 mm for CNN3. While EDV, ESV, LVM, and EF were used as clinical indices.

Similar and detailed research to analyze the impact of the amount of data, quality of images, and influence of expert annotation on LV segmentation was executed in [92]. A US images dataset that is openly available was also introduced in this work. The dataset consists of an apical 4-chamber view of 500 patients and is called the Cardiac Acquisitions for Multistructure Ultrasound Segmentation (CAMUS) dataset. The authors compared the performance of different CNN models based on U-Net. The models used for LV segmentation were U-Net1, U-Net2, anatomically constrained neural network, stacked hourglasses, and U-Net++. All these architectures were based on encoder-decoder and the main difference among these architectures is the use of different layers and learning parameters. The U-Net2 yields the best segmentation results, and the performance was slightly better than U-Net1, but U-Net1 needs fewer parameters to learn, so the authors choose U-Net1 for further experiments. The model was trained to segment only LV and multistructure in which the model segments the LV endocardial (endo), LV epicardial (epi), and left atrium (LA). The model performance was consistent for both LV segmentation and LV segmentation in the context of LA.

The effect of image quality on training was also tested. Two different sets of images are given to the network for training. One set comprises only high-quality images while the other consists of high- and low-quality images. The output of both sets does not vary significantly. The author infers that the encoder-decoder-based techniques can cope with variability in image quality. The influence of the size of the training dataset on the performance was also tested. The U-Net1 model was trained by increasing the dataset from 50 patients to 400 patients. At each level, 50 more patients' data are added for network training. The results show that the performance of the model increases to 250 patients and slightly improved by increasing the training data further to 400. It is concluded that U-Net1 requires 250 patient data to attain a good promising result. The impact of expert annotation was evaluated by annotating the data by three different experts. The network was trained each time using the data of 50 patients labeled by every three different experts. The validation data were kept the same, and the model was tested by the remaining 400 patients' data. The network trained using the expert's data showed better results in testing. It is analyzed that the data contouring images are cardiologist dependent. Furthermore, the encoder-decoder network can learn a specific way of segmenting.

The labeling of large dataset problem was addressed in [93]. A model was proposed to generate the ground-truth images. Pseudoimages were generated using a graphical model such as the principal component analysis. The CycleGAN model was employed to generate the labeled images by using the pseudoimages and unlabeled original images. These labeled images were utilized to train a U-Net model. CAMUS dataset, EchoNet dataset, and synthetic dataset were used to train and test the model. The results show that the model trained using the synthetic data also performs very well.

#### 4.2.6. Other Models Based on U-Net Architectures

Segmentation of LV, RV, and MYO from apical 2 chamber (A2C) view or apical 4-chamber (A4C) view has been implemented using DL methods [94]. In this work, neural network was tested to segment the LV, RV, and MYO from the apical long axis view (ALAX). In ALAX the main difference is the LV outflow tract (LVOT) which restricts the view. Four different approaches were used in this research. First, U-Net1 was trained from scratch and used to segment the ALAX. This model was referred as a baseline model in this work. Secondly, the baseline network was trained on A2C/A4C views, used as a transfer learning, and then trained for ALAX segmentation. Third, the baseline network was trained using A2C, A4C, and ALAX data. As ALAX data are less than A2C/A4C, so to compensate for this, ALAX data were repeated ten times in each epoch. In the fourth approach, the network was fed with US images and binary indicators. The purpose of the binary indicator is to inform the network about the input image whether it is ALAX or A2C/A4C. As the U-Net model has no dense layer, so an image is created from a binary indicator and fed to the network. The dataset of CAMUS challenge consisting of 500 patients was used for training, while for ALAX view, separate data of 106 patients were collected. The proposed multiview segmentation network achieved the best DSC value of 0.921.

To achieve the accurate and precise LV boundary and size, different studies modify the U-Net to elevate its performance. Gutierrez-Castilla et al. [95] improved the U-Net model by applying the changes in skip connections. The features' maps from each decoder layer were selected and upsampled according to the size of the final decoder output. After upsampling each decoder feature map, all feature maps were concatenated or added together. Using these dense skip connections, the decoder can flow directly to the final layer from each decoder layer. As no extra layers or filter is added, so this model does not add any extra parameters. For training, the model two datasets ACDC and Sunnybrook were used which consist of 150 and 45 patients' data, respectively. LV, RV, and MYO were segmented for diastolic and systolic. DSC and HD were used as evaluation matrices. As a clinical index, EF was also calculated by segmenting the LV for ED and ES. For ED, 0.968 and 4.855 (mm) values of DSC and HD were achieved, respectively. Likewise, DSC of 0.944 and HD of 6.254(mm) were attained for ES.

In the same way, a CNN model, named batch-normalization-U-Net (BNU-Net) was designed for LV segmentation from MRI images [96]. The proposed model was based on U-Net architecture, where the successive layers in the encoding path were followed by an ELU as an activation function and batch normalization was applied after convolutional filters. The BNU-Net has 4 layers in the contraction path and 7 layers in the expansion path. The 2 × 2 Max pooling was used after a pair of convolutional layers in the contraction path. The model was also trained using the ReLU activation function and was found that the model gives better performance with the ELU activation function. The model was trained using the publicly available Sunnybrook dataset and the training data size was increased by applying the affine method for data augmentation. DSC and sensitivity matrices were used to compare the performance of BNU-Net with U-Net (with and without data augmentation). The BNU-Net performed better with data augmentation and gave a value of 0.93 for DSC and 0.97 for sensitivity.

Also, a novel U-Net-based method, CNN module, named the “OF feature aggregation network” (OF-Net) as integrated temporal information from cine MRI into LV segmentation [97]. The proposed model integrates the motion information with the U-Net model. Furthermore, two more CNN models were used to localize (ROI-Net) and then segment the LV (called attention module). The model is trained using a flying chair dataset and fine-tuned using the MRI datasets. Two different publicly available datasets, Statistical Atlases and Computational Modelling of the Heart (STACOM) and ACDC datasets, were used. Out of 100 subjects, 66 were used for training and 34 for testing. Total of 12,720 images for training and 6972 for testing (from the STACOM dataset). A DSC value of 94.8 ± 3.3 was achieved.

In [98], a graphical user interface is developed for LV segmentation from MRI images using PyQT libraries. Images were labeled manually, and the labeled LV images were fed to train the CNN model. A publicly available dataset and the internal dataset were used to train the model with 13,535 images and test the model with 4,148 images. The model achieved the DSC of 0.87 ± 0.02.

The sonographers also used the point-of-care ultrasound (POCUS), which is portable ultrasonography used for diagnosis. The feasibility of translating the POCUS echo images to the high-quality traditional echo images was studied in [99]. To improve the quality of POCUS data according to the level of cart-based US data, the mapping from POCUS images to cart-based US images was an obligatory task. To achieve this goal, the POCUS images were analyzed, compared, and mapped with the traditional US images. The dataset of 5000 POCUS images and 16000 US images was used for the mapping purpose. The anatomy of LV was extracted from POCUS (using A2C view) using the DL method and then mapped with high-quality US images. The images were classified as low quality (fair + medium) and high quality. This classification was performed based on the visibility of the anatomy of the desired region. Fully convolutional encoder-decoder networks based on U-Net architecture were utilized for the translation of images. The size of the input image was 128 × 128. The model comprises ten encodings and eight decoding convolutional layers. ReLU activation, batch normalization, and dropout with ratio = 0.2 were used. In the first layer, batch normalization was not employed. Max-pooling and transpose convolution layers with stride 2 × 2 were used in downsampling and expansive paths, respectively. The average DSC value obtained is 82.6 ± 12.3 and 88.3 ± 5.0 for low- and high-quality images, respectively. Similarly, 2.6 ± 2.7 and 1.9 ± 0.8 mm values of HD for low- and high-quality images.

Despite the several advantages of using the U-Net in medical images, it ignores the effects of features maps on different scales directly. To solve this problem, a pyramid network is combined with the dilated U-Net model and named as multifeature pyramid U-Net (MFP-UNet) [100]. In dilated U-Net model, two more downsampling layers were added to extract more dense details of an image. As the US images were usually low contrast images, the images were preprocessed to enhance the contrast of US images using Niblack's method for global thresholding. The model was trained using a self-collected dataset of 137 2D-US sequences which yields 1080 training images and 290 test images. Furthermore, the model was also trained using the publicly available CAMUS dataset. The proposed model did not only yield good segmentation results but also took less runtime. The model was compared with U-Net, dilated U-Net, and DeepLabv3. It takes about 1.2 sec for the classic U-Net, 1.33 sec for DeepLabv3, and 0.81 sec for MFP-UNet to segment a test image. DSC, HD, Jaccard distance, and mean absolute distance were used to compare the performance of the model.

Another concerning issue is, while computing the parameters, most of the DL models extract similar features at low levels. To avoid this problem, modification in the attenuation U-net model was proposed by introducing the attention gates mechanism [101]. This model focuses on the desired region of varying size and shape automatically. Furthermore, the class imbalance problem was addressed by introducing the Tversky loss. The model achieves 0.75, 0.87, and 0.92 for Jaccard index, sensitivity, and specificity, respectively.

One of the main problems which arise in DL architectures is gradient vanishing. The research [102] focuses on the gradient vanishing problem and proposed a model residual of residual (ROR) U-Net model. The encoding path of the proposed model is similar to ResNet-U-Net, but three shortcut levels are introduced in the ResNet-U-Net model. The First 3 × 3 convolutions and zero padding on the input image are applied. At the second level ResNet, the identity and convolutional blocks of ResNet are divided into three branches, while, at the third level, convolutional blocks and identity blocks are used. The proposed model was trained and tested using the Sunnybrook dataset and compared with U-Net and ResNet-Unet models. The 0.866, 0.926, 0.923, 0.120, and 0.945 of Jaccard index, DSC, precision, false positive rate (FPR), and recall are achieved by the model.

The study [103] used the unsupervised learning method to segment the LV, RV, and MYO. A U-Net model was trained using ACDC and tetralogy of fallot dataset (TOF) dataset on short-axis (SAX) view of MRI images. Then, using the transformation network, the model segment the LV, RV, and MYO from the SAX view. The model has never seen the SAX view of images before.

**Figure 8:**
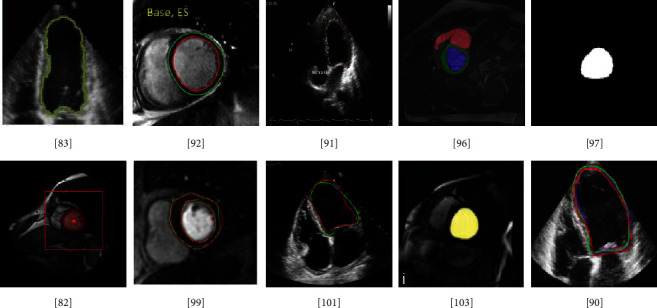
Sample of segmented images using U-Net.

In [104], the localization and detection of the area containing LV and RV were performed by a network known as the Left Ventricle Localization NET (LVLNET). This model is a lightweight encoder-decoder such as CNN. This CNN model contains two 3 × 3 kernels, batch normalization, and Max pooling in each layer having four layers in total. This localization model identifies the central part containing the LV and RV. The image is cropped and then pass to the next proposed CNN model called multigate dilated inception architecture (MGDIB). The MGDIB is based on U-Net architecture, but the kernel weights are expanded by the dilation factor and called a dilated convolution. The number of parameters does not increase using the dilated convolution. Two publicly available datasets, LV segmentation challenge (LVSC) + ACDC, were used. LVSC is used for the first part for localization while ACDC is for the segmentation of LV and RV. Different clinical measurements such as EDV, ESV, EF, and LVM are also calculated. DSC and HD values obtained were 0.900 and 0.910 for ED and ES, while HD values were 8.330 and 11.040 for ED and ES.  Figure 8 depicts instances of segmented images by the U-Net model.

### 4.3. LV Segmentation Using Other CNN Networks

Several studies also used various CNN models other than FCN and U-Net. The detail of other CNN models utilized for LV segmentation is explained in this section.

#### 4.3.1. CNN Models with Preprocessing

US imaging was also used for the heart analysis of children. For this analysis, the segmentation of LV and LA was performed on the paediatric US images [105]. Preprocessing was applied to the images before the training. The meaningless background was removed by resizing the images to 512 × 512. Furthermore, image augmentation was also used by rotating, random cropping, using salt and paper, and speckle noise with a probability of 0.01. To extract the spatial features, a spatial path module was designed. The spatial path is a convolutional network consisting of three layers with stride = 2, followed by batch normalization and ReLU activation function. It extracts a large amount of low-level spatial information. A submodule spatial attention was added to exploit the interspatial information and it focused on the “where” information part. The second parallel part of the network extracts the contexture information using five convolutional layers. RestNet50 was used as a backbone network. The submodule contextual attention was used at the end of this part to refine the extracted information and to know the “what” information part in an image is important. The context attention submodule focuses on “what” to look at, whereas the spatial attention submodule focuses on “where.” The authors compare the results of each submodule and conclude that “where” is more important in LV segmentation rather than focusing on “what.” In the last part, the features of both the parts are fed into another module called feature fusion module that generates the final refine features. A self-collected dataset of 127 4CH videos (100 for training and 27 for testing) was used. Images were extracted from the videos and 3654 images were used for training and 831 for testing. The segmentation results were evaluated step by step by adding each model, and finally, full Attention-guided Dual-path Network shows the best result. Additionally, well-known CNN architectures (FCN, U-Net, DeepLab, PSP Net, and BiSeNet) were also trained and results were compared with the proposed network. The DSC coefficient achieved a score of 0.951 and 0.914, in the LV and atrium segments, respectively.

#### 4.3.2. Hybrid Segmentation Methods

Some researchers analyzed the performance of hybrid CNN models for LV segmentation. The morphological models, snake models, and active shape model (ASM) were combined with CNN models in different studies. A hybrid model using DL and morphological methods was employed in [106]. The ROI was detected using a CNN model; then, LV was segmented using a multiscope CNN model. Three different patches were used for training the network, i.e., 8 × 8, 16 × 16, and 24 × 24, covering different scopes. Finally, morphological filtering was used to refine the boundary segmented by CNN. The LUMC dataset was utilized for this work. The model achieved the DSC value of 0.71.

Another hybrid approach based on CNN and the double snake model was proposed to segment the LV from MRI images [107]. A SegNet architecture was used for the initial segmentation result. Then, ROI was plotted around the coarsely segmented region taking the center point of the segment object and rectangular ROI was formed using polar transform. Output from SegNet was fed to the snake models which perform the final segmentation of LV. For training, the model 45 subjects were used and accessed from the MICCAI challenge. The DSC value of 0.96 and 0.97 was achieved for endo and epi. EF and LVM were calculated as clinical indices. Additionally, regression and Bland–Altman analysis was also performed.

Similarly, the work in [108] combines the ASM and neural network to segment the LV. A diffusion filter was applied to the images as a preprocessing step before feeding the data to the model. This filter used eight neighboring edges to preserve the edge information along with noise removal. A CNN architecture, Faster-RCNN, was used to determine the position of LV, and ASM used this location to segment the LV. As the ASM needs the initial position of the object to determine the position, so the region proposal network (RPN) was used to propose the regions that might contain the LV. Then, Faster-RCNN located the LV in the proposed ROI. Both RPN and Faster-RCNN were fine-tuned with ImageNet. The dataset of 30 patients was used for this work. The DSC, MAD, and HD were used to evaluate the performance. Furthermore, the models were also compared with other models proposed in the literature. The proposed model yields a DSC value of 0.921, MAD 1.95, HD 6.29 mm, and Jaccard 0.86.

One more hybrid approach was proposed consisting of the CNN model and dynamic programming [109]. Initially, SegNet [110] with 17 stacked convolution layers was used for coarse segmentation which segments the boundaries of LV. The batch normalization, ReLU activation function, and four MaxPool layers were used after the first four convolution layers. Secondly, the segmented results from SegNet were refined for endocardial contour. In the last step, a dynamic programming model was used to calculate the epicardium and endocardium of the heart. The 900 subjects from Hubei hospitals were used for this study. Jaccard and DSC were used for the evaluation. The DSC of 0.90 (0.03) and 0.93 (0.02) were obtained for endo and epi, respectively. Similarly, 0.80 ± 0.06 and 0.76 ± 0.09 average value of Jaccard was obtained. The LV-EDV, LV-ESV, SV, EF, LVM in the diastolic phase, and LVM in the systolic phase were also measured. The Bland–Altman analysis was performed for the comparison of these clinical indices.

#### 4.3.3. CNN + LSTM

A combination of encoder-decoder network and LSTM was used in [111] with Fire dilated and D-Fire dilated layers as a replacement for standard convolutional layers. The Fire dilated modules add an extra dilation rate in the kernel by inserting zeros between the consecutive values of the kernel and skip connections were applied to keep the temporal information of the image. Using the Fire dilated module, the network extracted more image information by adding extra parameters. Between the encoder and decoder, an LSTM module is added which is a special RNN structure. LSTM along with propagating the characteristics also captures the temporal dependencies between consecutive frames. Images were preprocessed by cropping the image based on ROI and resized to 80 × 80. A total of 2900 images from 145 subjects were used to evaluate the performance of the model and two experts manually labeled the images. DSC, Jaccard distance, accuracy, and positive predictive value (PPV) were used as evaluation metrics. The model achieved the DSC 0.960, Jaccard 0.903, accuracy 0.991, and PPV of 0.960 for LV. The proposed model was compared with simple Conv-Deconv, SegNet, FCN, and U-Net architectures.

A segmentation-based deep multitask regression learning model (Indices-JSQ) was proposed in [112]. The model is mainly divided into two parts. The first part is a segmentation network named Img2Contour and the second part is a multitask regression model (Contour2Indices). The segmentation model is based on deep convolutional encoder-decoder architecture with three convolution layers. The ReLU activation function along with Max-pooling was employed. Feature maps were generated by the use of the convolution layers with the kernel size of 5 × 5. This part segmented the LV and then passed the information to the next part of the model. The second part consists of RNN with LSTM. Three parallel CNN architectures were used that differ in kernel size and pool size. For the 1st CNN model, kernel size and pool size were 3 × 3 and 2 × 2, the 2nd model was 3 × 3 and 5 × 5, and the 3rd model have the same size of kernel and pool, i.e., 5 × 5. The dropout layer was used to avoid the overfitting problem. Information was passed to the LSTM which further quantifies the indices. A total of 2900 short-axis views of 145 subjects were used for training. DSC and mean absolute error (MAE) were used for the performance evaluation, and the performance is compared with other CNN models. Area, dimension, and wall thickness were also calculated as clinical indices. The proposed model automatically calculates these indices which is one of the major contributions of the model.

The tumor extraction using the convolutional LSTM network was performed in [113]. To prove the generalization of the proposed ST-ConvLSTM model, it was applied on 4D ultrasound for LV segmentation. The model was trained on the publicly available 3D + time ultrasound dataset challenge on Endocardial Three-dimensional Ultrasound Segmentation (CETUS) consisting of data from 15 patients. The proposed model achieved the DSC of 0.868 and 0.859 for ED and ES phases, respectively.

The classification and segmentation of LV from multiview (A2C, A3C, A4C) US images were implemented in [114]. Initially, pyramid dilated dense convolution (PDDConv) was used to extract multilevel and multiscale features. PDDConv network consists of batch normalization, ReLU, and dilated convolution. After extracting the features, hierarchical convolutional layers with LSTM recurrent units (hConvLSTM) were used for segmentation. The fully connected layers were used to perform the classification task using 3DCNN. Data of three different views, i.e., A2C, A3C, and A4C with 150 patients for each view yielding a total of 450 patients' data consisting of 13,500 frames, were utilized for training and testing. Furthermore, the model was trained and tested using the publicly available CAMUS dataset which has 1800 frames. To evaluate the performance of the models, MAD, DSC, and HD matrices were used. DSC of 0.92 for all A2C, A3C, and A4C views was obtained. The mean HD of 6.06 mm, 5.96 mm, and 6.06 and mean MAD of 2.80 mm, 2.77 mm, and 2.83 were achieved for A2C, A3C, and A4C views, respectively. The proposed model was compared with U-Net, ACNN, and U-Net++ and achieved better results. The EDV, ESV, and EF for the CAMUS dataset were also estimated using the segmented LV.

#### 4.3.4. Alternative CNN Models

A DL model was proposed to segment the LV and calculate such as cavity area, MYO area, cavity dimension, and wall thickness [115]. The model is named cascaded segmentation and regression network (CSRNet) and has two parts: a CNN model that segments the LV and a regression model to quantify the LV metrics. The dense connected convolutional neural network (DenseNet) was employed to reduce the number of learning parameters. The network mainly consists of three dense and three transition blocks. It generates three different probability maps for background, MYO, and cavity. Output from the last layer was fed to the regression component and passes to a CNN model with three convolution layers and two fully connected layers. To train the network, 2900 images (145 subjects) were used. These images were parted into 2320 training and 580 for testing. Several preprocessing methods such as landmark labeling, rotation, ROI cropping, and resizing were also applied to the images. DSC is calculated and compared with U-Net. The performance of DenseNet was better and achieved 0.989, 0.886, and 0.959 for background, MYO, and cavity. Similarly, the number of parameters, training, and testing time of CSRNet were compared with U-Net and DenseNet. The CSRNet has 0.6 M learning parameters with 17.62 training time and 1.20 sec testing time in 100 epochs.

A good initialization is a key parameter that optimizes the CNN model quickly. In [116], an initialization method was designed for the DCNN model to segment LV using MRI images. The model was trained and tested using two initialization methods: random initialization and Gabor filter initialization. Gabor filters can provide an accurate description of most spatial characteristics of simple receptive fields. Furthermore, spectral and spatial domains were simultaneously optimized in these filters which minimized the number of features. The authors demonstrated that using Gabor filter initialization requires less amount of training data and less complexity due to lower parameters. The York Cardiac Segmentation database (5011 images) was used for training. The model achieved the DSC of 0.798 with random initialization and 0.80 with Gabor initialization, while if Gabor filtered was maintained, the value further increased to 0.803.

A dense V-Net model was proposed which is based on V-Net architecture [117]. Few dense layers were added to the original V-Net model to improve the performance. For training, 30 patients' data (86 frames and each frame containing 73 images) were collected and manually labeled by 3 experts. The improvement of the proposed model was shown by comparing it with U-Net, FCN, and V-Net. The proposed model achieved a DSC of 0.90.

A transformers-based [5] DL model was designed to handle the sequential data. Transformers were mainly used for natural language processing and in [118] it is used to learn the image parameters. In the first part, 3D LV volume was passed to the transformer net which consists of 3D Conv layers, batch normalization, ReLU, Max-pooling layers, and fully connected layers. These layers extracted the transform parameters from the LVV and were inserted into AtlasNet, a new shape generation framework. The Atlas network has several advantages such as improved precision and generalization capabilities, and the possibility to generate a shape of arbitrary resolution without memory issues. AtlasNet consists of deformable layers and generates the 3D LV shape using the parameters achieved from the transformer. DSC, MSD, and HD were used for evaluation and achieved 0.91 ± 0.027, 1.99 ± 0.64, and 8.92 ± 7.16 respectively.

**Figure 9:**
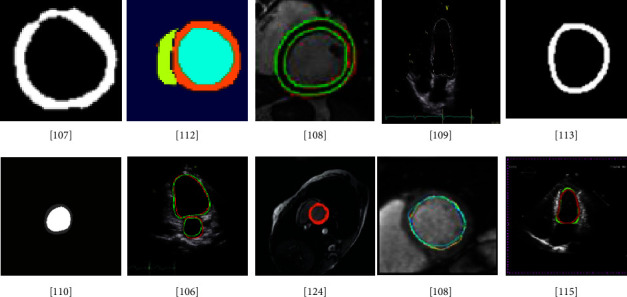
Sample of segmented images using other CNN models.

The CNN models such as FCN and U-Net focused on single-frame image processing. While, in the study [119], a dense RNN was proposed to segment the LV from a four-chamber view of the MRI time sequence. RNN can deal with sequential information. In RNN, information from the previous cell was transmitted to the next LSTM cell, but the first cell does not get any previous information. The proposed model used the two RNN models. The first layer of the second RNN model, which performs the segmentation, receives the information from the first RNN model. In this experiment, data from 137 patients were used. The performance of the model was compared with state-of-the-art CNN models. The proposed model achieved the IoU of 92.13%. Few examples of the segmented LV by CNN models are depicted in Figure 9.

## 5. Discussion

The performance of DL methods depends on various parameters, and the time for the data processing is based on hardware. The details of the several modified models and proposed architectures are explained in Section 4. Here, in this section, some important data from the reviewed literature are presented. The section is divided into five sections and conveyed in a tabular form so that readers can have an overview of all important information related to hardware, software, imaging modality, database, architecture, and results.

### 5.1. Imaging Modality

For the analysis of cardiac diseases, different imaging modalities have been used such as the US, CT scan, and MRI. Due to its high resolution, MRI is the gold standard and is mostly used. On the contrary, US images are also highly recommended due to their ease of use and low cost. The third type of image used for the cardiac analysis is CT.

### 5.2. Architectures

Several CNN architectures are used by researchers for LV segmentation. The U-Net architecture is specifically designed for medical images; therefore, the use of U-Net and its variants are mostly used for the segmentation of LV. Besides U-Net architecture, FCN is the second most used network architecture for LV segmentation.  Table 1 shows the CNN architectures used for LV segmentation.

### 5.3. Hardware

During the training process, the neural networks learn millions of weights. It may take several days to train such a huge number of weights on CPUs. The training time taken by the machine is one of the parameters to be focused on while implementing the models. Therefore, for the processing of DL models, hardware configuration plays an important role. A striking option for DL is a GPU. The use of GPUs makes the training and testing process fast, and results can be attained and compared in a short time. Hardware configurations used by authors for LV segmentation are listed in Table 2.

### 5.4. Software

An appropriate software framework is necessary to execute the complex DL architectures. Various frameworks have been used to implement the LV segmentation through DL architectures. These frameworks are generally used in Python programming. Python is an open-source programming language; furthermore, it supports a remarkable set of easy to utilize library functions for the execution of DL models; therefore, Python is widely used in DL-based applications. The software frameworks described in this section are primarily developed in Python language. The most general among them are TensorFlow, Theano, Keras, CAFFE, Torch, and Deeplearning4j. Few researchers have also used MATLAB as a programming language. Software used by researchers is enlisted in Table 3.

### 5.5. Dataset

The performance of DL models is highly affected by the dataset. The number of images or number of patient data used to train and test the model is one of the key attributes of LV segmentation. Most researchers have used self-collected data, but, at the same time, several public datasets are also available. The details of the datasets are explained below and summarized in Table 4.

Data are from the 2009 Cardiac MR Left Ventricle Segmentation Challenge, often known as the Sunnybrook Cardiac Data. The data collection includes 45 cine-MRI images from a variety of different people and pathologies.

After registering on a website dedicated to the online evaluation, the ACDC database is made accessible to participants through two datasets. One dataset, referred to as the training dataset, contains 100 patients and manual references based on the study of one clinical expert. Second is a testing dataset consisting of fifty additional cases without manual annotations.

The MICCAI 2009 database contains 45 samples of short-axis cine MR images, 15 training cases, 15 test cases, and 15 online cases, which are randomly divided. The MICCAI 2018 challenge dataset comprises 145 participants' processed CMR sequences. The ages of the individuals range from 16 to 97, with an average of 58.9 years.

The CAMUS dataset includes clinical exams performed on 500 patients at the University Hospital of St. Etienne (France). The LVSC dataset is accessible to the general public as part of the 2011 STACOM short-axis cine MRI semiautomatic LV myocardial segmentation challenge. This dataset is comprised of 200 individuals with myocardial infarction and coronary artery disease.

The short-axis steady-state free precession cine MRI from the Cardiac Atlas Project database is used to make up the STACOM dataset. In total, 100 individuals with postmyocardial infarction and coronary artery disease are included in the dataset. Every image contains a ground truth annotation.

The TWINS-UK is a volunteer register consisting of more than 12,000 twins. One thousand four hundred and sixty eight consecutive female volunteers (mean age 62 9 years) were recruited for this investigation. Each dataset had 12 to 14 short-axis cine that were continuous and evenly spaced from the atrioventricular (AV) ring to the apex, covering both ventricles.

The UKBB dataset is comprised mostly of a large number of healthy volunteers. By stacking a series of 2D cine images, 3D images of the LV and RV were created. LV, MYO, and RV were manually segmented in the ES and ED phases by 8 observers under the direction of 3 lead investigators, and hundred subjects were chosen.

**Figure 10:**
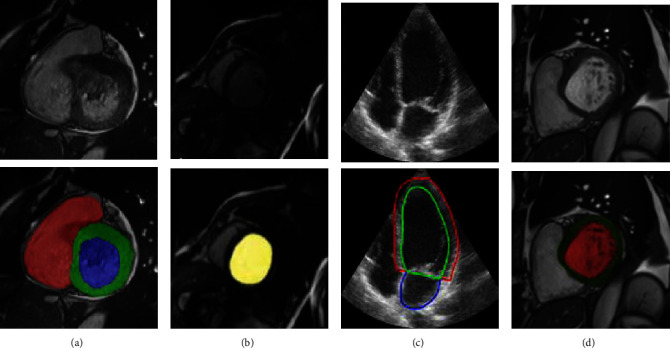
Example of the original image (top) and ground truth (bottom) of (a) ACDC, (b) Sunnybrook, (c) CAMUS, and (d) MICCAI datasets.

The Cardiac Atlas Project offers CMR for 95 individuals with coronary artery disease and mild-to-moderate left ventricular dysfunction from prospective, multicenter, and randomised clinical studies. Sufficient slices along the short axis were collected to cover the whole heart in SAX. Also included in these acquisitions was the manual segmentation of the myocardium. The CETUS dataset came from 15 patients. Each patient had 13–46 3D volumetric imaging sequences, and each sequence had two manually segmented volumes at the end-diastole (ED) and end-systole (ES) phases.  Figure 10 is an illustration of original and labeled image taken from four distinct datasets.

### 5.6. Results

The segmentation performance of models is evaluated using well-known evaluation matrices such as DSC, HD, and Jaccard distance, although some authors also used other matrices for accuracy, sensitivity, etc.

The DSC [123] is overlap based and calculated using equation (1). In the equation, *S*_GT_ represents the ground-truth image that represents the original LV size and boundary. *S*_Seg_ is the segmented mask by the model. To calculate the DSC, the intersection region of two masks is divided by the total region of both masks. The range of DSC is 0 and 1, where 0 represents no similarity or overlap and 1 represents exact overlap:

(4) $$\begin{array}{c} DSC=\frac{2\left|S_{GT}\cap S_{Seg}\right|}{\left|S_{GT}\right|+\left|S_{Seg}\right|}\text{.} \end{array}$$

The HD [124] is a spatial distance-based index to measure the “closeness” of two sets of points. The HD between two-point sets *A* and *B* is defined by equation (2).

(5) $$\begin{array}{c} d_{H}\left(A,B\right)=\max\left(h\left(A,B\right),h\left(B,A\right)\right)\text{,} \end{array}$$

where *h* (*A*, *B*) is direct Hausdorff distance, and it can be calculated by equation (3).

(6) $$\begin{array}{c} h\left(A,B\right)=\max_{a\epsilon A}\min_{b\in B}\left\|a-b\right\|, \end{array}$$

where ‖*a* − *b*‖ is any norm value, e.g., Euclidean distance.

The Jaccard distance [123] can be calculated using the formula presented in equation (4).

(7) $$\begin{array}{c} J=\frac{\left|X\cap Y\right|}{\left|X\;U\;Y\right|}\text{,} \end{array}$$

where *X* and *Y* are the ground-truth and segmented output images, respectively.

Similarly, the precision, specificity, and IoU can be computed with the use of equations (5)–(7), respectively:

(8) $$\begin{array}{c} Accuracy=\frac{TP+TN}{TP+TN+FP+FN}\text{,}\\ Specificity=\frac{TP}{TP+FP}\text{,}\\ IoU=\frac{overlapping\;area}{combined\;area}\text{.} \end{array}$$

The assessment matrices and theoretical values attained by researchers are shown in Table 5.

## 6. Challenges and Future Outlook

The article shows that DL approaches have equally performed or outperformed the previous state-of-the-art LV segmentation techniques. DL algorithms are expected to completely replace the current LV segmentation techniques. Given this, it is reasonable to consider whether DL techniques can be directly applied to real-world applications to reduce medical practitioners' workload. However, there are still challenges to make the existing DL methods viable for real-time applications.

In medical images and, particularly, cardiac images, acquiring the annotated images is the most prevalent challenge. As this article demonstrates, most of the research employed supervised learning, which necessitates the usage of a significant number of annotated images. To properly label, the LV needs both specialised knowledge and a significant investment of time. As a result, the datasets of the annotated LV are quite limited in comparison to other publicly available datasets in other fields, such as natural images.

Moreover, the performance of DL on data that differs from the training dataset is another challenge. Even though the trained DL model is tested on unseen data, the training and testing data are received from the same source, such as the same sort of scanner. The model does not provide the anticipated outcome if new types of data, e.g., from multiple scanners or different disease patients, are used to test the model. A few studies have utilized training data for LV segmentation from different sources and scanners to train the model to get over this problem.

Also, the DL performance is highly dependent on the quality of the training images. Many imaging modalities such as CT and US are of low quality due to many factors such as speckle noise and poor contrast ratio. To produce high-quality images, many researchers use some sort of data preprocessing.

Therefore, further studies are required to investigate the methods to improve the image quality. Therefore, the efficiency of the DL model and the accuracy of LV segmentation may be significantly boosted by improving the image quality. There is a significant demand for a DL-based system that has the ability to improve image quality in an efficient and effective manner while simultaneously reducing noise. Therefore, the LV segmentation will be considerably more accurate when the segmentation and enhancing methods are combined.

In addition, the integration of LV segmentation algorithms with additional patient data, such as patient history, age, and demographics, is an important area of the article that might further improve the performance of clinical decision making and assist physicians in calculating clinical indices.

As discussed above, one of the main challenges is the availability of large datasets, and there is abundant new research aimed at levitating the limited dataset size problem, and some LV datasets are publicly available. There is a pressing need for architectures and algorithms that have been purposed and built for the segmentation of medical images and, therefore, LVs, and that can also perform admirably when applied to limited datasets.

## 7. Conclusion

In this article, a comprehensive review of the literature focused on the analysis of cardiac images using DL for LV segmentation is presented. In the field of image processing CNN, a subbranch of DL has shown very promising results for different types of identification including classification, object detection, and segmentation. CNN is also seen as a futuristic approach specifically in image processing. The application of CNN in medical images is extensive. Therefore, this work details and summarizes the uses of CNN for LV segmentation. The most common imaging modalities (MRI, US, and CT scan) were briefly introduced in the article. The basics of CNN architectures were also discussed to have a better understanding of these models. Among the different CNN models, FCN, U-Net, and modified model two are mostly used for LV segmentation. This work also gives a detailed discussion of hardware, software, and dataset used for LV segmentation. The different evaluation matrices used for the performance analysis of the models were also discussed. A comparative summary was tabulated to ease the comparison for the readers. This work lays a foundation for the readers for an instinctive understanding of DL methods used for LV segmentation specifically for medical and cardiac images.

## Figures and Tables

**Table 1:** Studies used different CNN models for LV segmentation.

Architecture	Study
FCN	[38, 49, 49, 57, 61–63, 66, 68, 69, 75]
U-Net	[77–86, 88–104, 120]
Other CNN models	[64, 105–109, 111–119, 121, 122]

**Table 2:** Details of hardware for LV segmentation.

Study	Hardware
[75]	Nvidia Tesla K80 GPU
[63]	CPU: Intel 4790k, GPU: NVIDIA TitanX
[66]	Intel Xeon(R) E5-2640 CPU @ 2.60 GHz, NVIDIA Tesla K40c GPU, 128 GB RAM
[38]	Intel (R) Core (TM) i7-6700 CPU @ 3.40 GHz with 4 cores and 32 GB RAM; the graphics processing unit used was an Nvidia GTX 1080Ti model with 11 GB RAM and 3584 CUDA cores
[69]	Intel (R) Xeon (R) processor ES-1650 at 3.50 GHz with 12 cores; Nvidia Quadro K4200 model with 4 GB of RAM and 1440CUDA cores
[82]	NVIDIA DIGITS DevBox
[83]	Intel i7-6700 CPU and an NVIDIA GeForce GTX 980M GPU
[84]	Nvidia GTX 1070 GPU
[85]	3.4 GHz Core i7 CPU, 64 GB RAM, Nvidia TiTan X (12 GB memories)
[91]	GeForce GTX 1080; Nvidia, Santa Clara, Calif
[90]	Nvidia GTX 1080Ti
[95]	Titan Xp GPU donation from NVIDIA Corporation
[96]	NVIDIA GeForce Titan X Pascal GPU
[81]	NVidia GTX 1080 Ti (12 GB)
[120]	Nvidia 11 GM RAM
[98]	8 GB GPU (NVIDIA GeForce GTX 1080).
[92]	Nvidia Tesla M60 GPUs (8 G RAM).
[100]	12 GB of RAM, a GPU-based graphic card with 2496 CUDA cores (Tesla K80), and an Intel Xeon CPU.
[77]	(Intel(R) CPU i7-7770K, 4.2 GHz, 16G RAM) with an NVIDIA GPU (GeForce, GTX TITAN X, NVIDIA Corp., Santa Clara, CA, USA)
[78]	Two Intel Xeon 8 core CPUs, 12 GB of RAM, and an NVIDIA Quadro P6000 GPU
[88]	Four NVIDIA GTX 2080Ti GPU cards, each with 11 GB RAM
[101]	3 Nvidia GTX 1080 Ti GPU
[80]	NVIDIA Titan GPU
[104]	NVIDIA GeForce GTX 1080 Ti GPU
[111]	Intel Core i5-7400 CPU. The graphics card is an NVIDIA GeForce GTX 1060
[115]	GeForce GTX 1080 ti GPU
[107]	Pentium dual-core 2.60 GHz hardware
[108]	CPU of AMD Phenom II X6 1055T Processor 2.8 GHz, 8G RAM, and VGA card of NVIDIA GeForce GTX 960 (CUDA v6.5)
[64]	GeForce GTX 1050 (4 GB GDDR5 dedicated) on an Intel Core i7-7700HQ (2.8 GHz, 6 MB cache, 4 cores) computer with 16 GB DDR4-2400 SDRAM
[105]	GTX 1080Ti graphic processor
[121]	GTX 1080Ti graphic processor
[117]	GTX 1080Ti graphic processor
[118]	NVIDIA Titan X GPU on Dell T7920 (GPU is Core I7, and memory size is 24 GB)
[113]	DELL TOWER 7910 workstation with 2.40 GHz Xeon E5-2620 v3 CPU, 32 GB RAM, and an Nvidia TITAN X Pascal GPU of 12 GB of memory
[114]	Two Intel Xeon 2.10 GHz CPU and four 12 GB Nvidia Titan XP GPU
[122]	GTX 1080Ti
[119]	NVIDIA Tesla P100

**Table 3:** Detail of software used for LV segmentation.

Study	Software
[57]	TensorFlow
[75]	TensorFlow
[63]	Not reported
[66]	Keras
[38]	MATLAB R2015b
[69]	Café
[82]	Keras
[83]	TensorFlow
[84]	TensorFlow
[85]	Café
[91]	TensorFlow
[81]	Keras
[120]	MATLAB 2019a
[98]	TensorFlow
[92]	Python/Keras
[100]	TensorFlow r1.12 and Keras 2.2.4.
[86]	Keras
[77]	MATLAB 2013a
[99]	Python with Keras-TensorFlow
[78]	TensorFlow/Ubuntu 18.04
[88]	Pytorch/Ubuntu18.04
[101]	Keras
[102]	Keras
[80]	MATLAB R2020
[89]	Keras
[106]	TensorFlow
[111]	Keras
[115]	TensorFlow
[107]	MATLAB
[108]	MATLAB *R* 2016b
[64]	TensorFlow
[105]	TensorFlow
[121]	TensorFlow
[116]	MatConvNet, an open-source library in MATLAB
[117]	TensorFlow
[118]	Pytorch
[113]	TensorFlow
[114]	TensorFlow
[122]	Anaconda 5.0.1 (python 3.5), TensorFlow, and Tensorlayer environment
[119]	PyCharm

**Table 4:** The detail of datasets used for LV segmentation.

Study	Dataset
[63, 68, 69, 81, 95, 96, 102],	Sunnybrook
[57]	TWINS-UK
[61, 66, 77, 95, 97, 103, 104, 120],	ACDC
[38, 61, 66, 85, 86, 107, 121],	MICCAI
[92, 94, 100, 114]	CAMUS
[101, 104]	LVSC
[97, 101]	STACOM
[77]	(UKBB)
[81]	Cardiac Atlas
[113]	CETUS
[103]	TOF
[62]	Own dataset (220 subjects)
[75]	Own dataset (5008 subjects and 93,500 images)
[61]	Own dataset
[49]	Own dataset 1100 subjects
[49]	Own dataset 1100 subjects
[82]	Own dataset (69 images)
[83]	Own dataset (500 patients)
[84]	Own dataset (250 patients)
[91]	Own dataset (596 subjects)
[90]	Own dataset (94 cases)
[98]	York + own dataset (17,683 images)
[100]	Own dataset (1080 + 290) +
[99]	Own dataset (5000 Pocus + 16000 cart base)
[78]	Own dataset: 492 scans
[88]	Own dataset 25,027 scans (*N* = 12,984 patients)
[79]	Own dataset 23 sequences (670 images)
[93]	Four different datasets
[80]	Kaggle, 484 examinations
[89]	Own dataset 563 patients
[106]	143 postinfarction patients, LUMC
[111]	Own dataset (2900 2D short-axis cine MR images of 145 subjects)
[115]	Own dataset (2900 2D short-axis cine MR images of 145 subjects)
[108]	Own dataset
[64]	Own dataset (30 scans and 2000 images)
[112]	Own dataset (2900 short-axis cardiac MR images of 145 subjects)
[109]	Own dataset (900 cases)
[105]	Own dataset (127 videos)
[116]	York cardiac segmentation database
[117]	Own dataset (30 patients)
[118]	Own dataset (50 scans)
[122]	Cap a total of 2490 images from 83 subjects
[119]	Own dataset 137 patients

**Table 5:** The details of results achieved for LV segmentation.

Study	Results
[57]	DSC (0.981 ± 0.025), APD (6.290 ± 8.381) mm, and conformity index (0.961 ± 0.0551)
[62]	DSC (0.934 ± 0.041)
[75]	DSC (0.94 ± 0.04), HD (3.16 ± 0.98), and MCD (0.35)
[63]	Volume estimation
[66]	DSC (0.963 ± 0.026) and (0.932 ± 0.075) and HD (5.58 ± 3.75) and (6.92 ± 4.69) for ED and ES, respectively
[61]	DSC (0.93 and 0.94) and HD (9.52 and 6.71) with cross-entropy loss and radial distance, respectively
[49]	DSC (0.883 ± 0.062), JD (0.795 ± 0.07), HD (13.4 ± 12.2) mm, MSD (1.0 ± 2.4) accuracy (0.883), sensitivity (0.921), and specificity (0.997)
[38]	APD (1.71)
[69]	DSC (0.93 ± 0.02) and (0.92 ± 0.01), APD (2.23 ± 0.31) and (2.13 ± 0.28) mm, and good contour (94.19 ± 7.38) and (95.64 ± 7.11) for endo and epi
[68]	DSC (0.93 ± 0.03) and APD (1.41 ± 0.24)
[49]	DSC (88.3 ± 6.2), Jaccard (79.5 ± 7.0), MSD (1.0 ± 2.4) mm, and HD (13.4 ± 12.2) mm
[82]	DSC (0.86 ± 0.06)
[83]	A bland-altman analysis mean difference of -13.7% and a standard deviation of 8.6% for EF
[84]	DSC (0.87 ± 0.03)
[85]	DSC (0.90 ± 0.05) and (0.81 ± 0.005) and HD (7.6 ± 4.5) and (0.91 ± 0.018) for endo and epi, respectively
[91]	Accuracy (0.93 ± .006) and (0.94 ± 0.005) for endo and epi
[90]	DSC (0.923 ± 0 : 03) and (0 : 924 ± 0 : 04) for ALAX and A2C/A4C
[95]	DSC (0.968) and (0.944) and HD (4.855) and (6.245) mm for ED and ES, respectively
[96]	DSC (0.93 ± 0.03) and sensitivity (0.97)
[81]	DSC (0.87 ± 0.0053), (0.869 ± 0.0051), and (0.868 ± 0.0047), MSE (0.0135 ± 0.0006), (0.0136 ± 0.0008), and (0.0138 ± 0.0007), and MAE (0.0137 ± 0.0006), (0.0138 ± 0.0008), and (0.0140 ± 0.0007) for U-net-BN-RL, uInception, and uXception, respectively
[120]	DSC (0.919) and IOU (0.860)
[97]	DSC (94.8 ± 3.3)
[98]	DSC (0.87 ± 0.02)
[92]	DSC (0.95 ± .023) and (0.95 ± 0.039), HD (5.3 ± 3.6) and (5.5 ± 3.8) mm, MSD (1.6 ± 1.3) and (1.6 ± 1.6) for ED and ES, respectively
[100]	DSC (0.94 ± 0.12), HD (1.62 ± .05), JD (0.98 ± .01), and MSD (1.32 ± .53)
[86]	DSC (0.893)
[77]	DSC (0.921), HD (3.9 mm), and ASSD (1.43 mm)
[99]	DSC (82.6 ± 12.3) and (88.3 ± 5.0) for low- and high-quality images, respectively
[78]	DSC (0.94)
[88]	DSC (0.87 ± 0.08) (3CS) and (0.82 ± 0.22) (2CS)
[101]	Jaccard (0.75), sensitivity (0.87), and specificity (0.92)
[102]	DSC (0.926), Jaccard (0.866), precision (0.87), FPR (0.120), and recall (0.945)
[103]	DSC (0.93 ± 0.04)
[79]	DSC (0.917), MAD (1.66 pixels), and overlap (85.57%)
[93]	
[80]	DSC (0.87 and 0.95) with and without transfer learning
[104]	DSC (0.900 and 0.901) and HD (8.330 and 11.040) for ED and ES
[94]	DSC (0.921)
[89]	DSC (93.6) and accuracy (97.7)
[106]	DSC (0.71 ± 0.09)
[111]	DSC (0.960 ± 0.008), JD (0.903 ± 0.026), accuracy (0.991 ± 0.005), and PPV (0.960 ± 0.040)
[115]	DSC (0.959) and HD (3.557),
[107]	DSC (0.97 and 0.96) for endo and epi
[108]	DSC (0.921 ± 1.87), JD (0.86 ± 0.007), and HD (6.29 ± 2.01 mm)
[64]	DSC (0.712 ± 0.031), sensitivity (0.881 ± 0.17), accuracy (0.968 ± 0.032), and specificity (0.978 ± 0.029)
[112]	DSC (0.87 ± 0.06)
[109]	DSC (0.90 ± 0.03) and (0.93 ± 0.02) and JD (0.80 ± 0.06) and (0.76 ± 0.09) for endo and epi
[105]	DSC (0.951 ± 0.031), accuracy (0.987 ± 0.007), precision (0.960 ± 0.050), specificity (0.996 ± 0.006), and sensitivity (0.950 ± 0.050)
[121]	DSC (0.803 ± 0.204), JD (0.706 ± 0.214), sensitivity (0.859 ± 0.2), specificity (0.998 ± 0.002) PPV (0.771 ± 0.206), and NPV (0.999 ± 0.001)
[116]	DSC (0.803), pixel accuracy (0.973), specificity (0.984), sensitivity (0.841), and mean accuracy (0.903)
[117]	DSC (0.9 ± 0.12)
[118]	DSC (0.91 ± 0.027), HD (8.92 ± 7.16), and MSD (1.99 ± 0.64)
[113]	DSC (0.868 ± 0.021) and (0.859 ± 0.016) for ES and ED, respectively
[114]	DSC (0.92 ± 0.04), HD (6.06 ± 2.11), (5.96 ± 2.07), and (6.06 ± 2.04) mm, MSD (2.80 ± 1.02), (2.77 ± 1.05), and (2.83 ± 1.04) mm for A2C, A3C, and A4C, respectively
[122]	Localization accuracy (6.45 ± 4.53 mm)
[119]	IoU (92.13%)

## Data Availability

All the data used to support the findings of the study are included within the article as references.
